# Space and scale in higher education: the glonacal agency heuristic revisited

**DOI:** 10.1007/s10734-022-00955-0

**Published:** 2022-11-17

**Authors:** Simon Marginson

**Affiliations:** 1 Department of Education, University of Oxford, 15 Norham Gardens, Oxford, OX2 6PY Oxfordshire UK; 2 Centre for the Study of Higher Education, University of Melbourne, Melbourne, Australia

**Keywords:** Higher education, Research, Spatiality, Geographical scale, Globalization, Global flows, International education, Glonacal

## Abstract

The 2002 ‘glonacal’ paper described higher education as a multi-scalar sector where individual and institutional agents have open possibilities and causation flows from any of the interacting local, national and global scales. None have permanent primacy: global activity is growing; the nation-state is crucial in policy, regulation and funding; and like the other scales, the local scale in higher education and knowledge is continually being remade and newly invented. The glonacal paper has been widely used in higher education studies, though single-scale nation-bound methods still have a strong hold. Drawing on insights from human geography and selected empirical studies, the present paper builds on the glonacal paper in a larger theorization of space and scale. It describes how material elements, imagination and social practices interact in making space, which is the sphere of social relations; it discusses multiplicity in higher education space and sameness/different tensions; and it takes further the investigation of one kind of constructed space in higher education, its heterogenous scales (national, local, regional, global etc.). The paper reviews the intersections between scales, especially between national and global, the ever-changing ordering of scales, and how agents in higher education mix and match scales. It also critiques ideas of fixed scalar primacy such as methodological nationalism and methodological globalism—influential in studies of higher education but radically limiting of what can be imagined and practised. Ideas matter. The single-scale visions and scale-driven universals must be cleared away to bring a fuller geography of higher education to life.

## Introduction

Two decades ago *Higher Education* published a paper by Simon Marginson and Gary Rhoades titled ‘Beyond national states, markets, and systems of higher education: a glonacal agency heuristic’, hereafter titled ‘the glonacal paper’. The authors^1^ aimed to ‘shape comparative higher education research with regard to globalization’ (Marginson & Rhoades, 2002, p. 282). The paper was written during an intense discussion about globalization, meaning worldwide integration and convergence in the economy and culture (Held et al., 1999). There was also unprecedented growth in cross-border activity in higher education and research, including international student mobility, collaboration in science, university partnerships and consortia, offshore branch campuses, and global ‘e-universities’, not to mention policy borrowing and the spread of Anglo-American institutional models. The Internet, founded in 1989, was expanding rapidly and emerging higher education systems were flooded with Westernizing information, knowledge, models and templates.

There were varying takes on globalization (e.g. Castells, 2000; Robertson, 1992; Sassen, 2002), but all saw it as geographical and spatial, highlighting global/local vectors, flat networks, time–space compression, and global flows as in Appadurai’s (1996) ethnoscapes, technoscapes, financescapes, mediascapes and ideoscapes. Along with others (e.g. Dale, 2005; Henry et al., 2001; Robertson, 2006; van der Wende, 2002), the glonacal paper proffered geo-cognitive space and scale as explanatory tools in higher education. ‘Glonacal’ referred to three interacting scales of activity and agency: *glonacal* = *glo*bal + *na*tional + lo*cal*. This new geo-spatial theory became commonplace in higher education studies (e.g. Enders, 2004; Horta, 2009; Komotar, 2021; Liu & Metcalfe, 2016; Oleksiyenko, 2019; Valimaa, 2004).^2^

The glonacal paper re-imagined higher education to take in a larger set of empirical objects and causal relations. ‘The aim of all theory is always the same: explanation and understanding’ (Mearman, 2005, p. 621). Geo-spatial theorizing can also open up a different world. Changes in the spatial theorization of higher education and knowledge can re-set the collective conditions of practice (consider the impact of the ‘global knowledge economy’ idea). There may never be consensus on the purposes of higher education, but there could be agreement on its multi-scalar ontology, which is one of the arguments made in the present paper. That would change what people see and do.

The paper reviews and substantially develops the 2002 theorization. In preparation, it has made use of a range of theoretical and empirical works from 1995 to 2022, not all of them listed in the references, on spatiality, globalization, higher education, scientific knowledge. It also draws on the author’s own research into global space and scale (Marginson, 2022d), on cross-border student mobility (e.g. Marginson et al., 2010) and on global and national science in higher education (e.g. Marginson, 2022a, 2022b, 2022c). Research science is integral and central to one part of higher education, the research-intensive universities. Though less than a fifth of R&D spending is in higher education (OECD, 2022), 85 per cent of published papers in the bibliometric collections that define global science (Marginson, 2022c) have at least one university author (Powell et al., 2017, pp. 2, 8–9).

Following this introduction, the next section reviews the glonacal paper, the evolution of higher education and knowledge since 2002 and whether the sector evolved as the glonacal paper expected. The following section theorizes space-making in higher education and knowledge, resting partly on Massey (2005) in human geography. Adapting Lefebvre (1991) and deploying examples from higher education, it argues that space-making combines the material, the imagination and social practices. The next section focuses on one kind of space-making in higher education and knowledge—geo-cognitive (material and imagined) scales such as national, global, regional and local. It expands on multi-scalar spatiality, including the heterogeneity of scales, intersections between scales, and questions of scalar primacy. These two sections are grounded in critical realism (Sayer, 2000) and social realism (Archer, 1995): they assume an open ontology of space and scale, in which neither social structure nor human agency can permanently fix the other. The conclusion follows.

## Glonacal 2002 and after

The glonacal paper was a product of its time. It had two starting points. First, cross-border activity was growing rapidly, but higher education studies lacked ‘a framework for conceptualising agencies and processes that extend beyond the nation-state’ (Marginson & Rhoades, 2002, p. 285). The standard national system model, with embedded local institutions plus international activity at the edges of the system, could not fully grasp either the global or the local, and something more was needed. Second, the authors pushed back against the then-widespread belief that national and global power were zero-sum and the advance of globalization meant the weakening of the nation-state. Yes, higher education institutions were affected by growing global and regional exchange and by pan-national organizations like the World Bank and OECD. Many universities were ‘global actors’ in their own right (p. 282). But the binary of global/local that dominated accounts of globalization, as if nations were being eclipsed, was clearly insufficient to explain higher education. Nation-states continued to define, regulate and fund the sector. Global flows were often refracted by nation-states. The glonacal theorization put nation-states back in the picture.

It also argued that because institutions were ‘globally, nationally and locally implicated’ (Marginson & Rhoades, 2002, p. 288), no one scale could be always determining. ‘We do not see a linear flow from the global to the local; rather we see simultaneity of flows’ and reciprocal effects (p. 292). Causation could be generated in any scale, and ‘at every level – global, national, and local – elements and influences of other levels are present’ (pp. 289–290). The field needed a multi-scalar framework that could empirically track higher education in all three ‘planes of existence’ together (p. 281). This was the purpose of the glonacal agency heuristic. The word ‘agency’, inserted by Rhoades, was significant. Global, national and local higher education was more than structures. It included active agents who moved across different scales. Activity was shaped by history and material resources, ‘layers and conditions’ (p. 291), by the national and global stratification of higher education (p. 301; Marginson, 2006) and also by the vision, imaginings and discourses of agents. Globalization in higher education—just like national systems of higher education and like all kinds of local activity—was deliberately created by persons, organizations and governments.

The glonacal paper endorsed Deem’s (2001) proposal for ‘situated case studies’ to map spatial flows and effects. Held et al. (1999) had shown that globalization varied sector by sector, and Marginson and Rhoades (2002) highlighted the autonomy of space and scale in higher education. Commentators from both left and right identified globalization in higher education with the annexation of the sector by an all-powerful and de-territorialized global knowledge economy and with neoliberal regulation by nation-states. But this picture reified economic globalization while leaving the old nation-boundedness undisturbed. ‘The global … is invoked as a residual explanation for observed commonalities across countries’ (p. 285). For example, Torres and Schugurensky (2002) argued that a common denominator of change in worldwide higher education was a reduction of institutional autonomy driven by globalization. In contrast, Marginson and Rhoades argued for a mixed picture: in some global activities, autonomy was enhanced. In any case, institutions were complex entities with multiple inner and outer drivers. They were more than branches of the economy.

The metaphor of academic capitalism reveals a powerful global trend but blinds us to the power of national traditions, agencies, and agents in shaping the work of higher education, as well as to the local agency exercised by students, faculty, non-faculty professionals, and administrators, pursuing prestige, knowledge, social critique, and social justice (Marginson & Rhoades, 2002, p. 287).

### Reception of the glonacal paper

In higher education studies, the core of the glonacal heuristic, the three simultaneous dimensions of activity, has not been challenged. Some scholars have made it more complex. Jones (2008) notes that the local scale embodies disciplines as well as institutions, and epistemic communities have differing patterns of global engagement. For Komotar (2021), the local scale encompasses all institutions, disciplines and individuals. Robertson et al. (2016) discuss regionalisms. The regional scale is a primary scale in higher education in Europe, given the Bologna structural reforms, Erasmus student mobility, European research funding and collaboration, and U-Multirank, and it is also significant in Southeast Asia (Chou & Ravinet, 2017).

Naidoo (2010) highlights a more fundamental limitation of the glonacal paper:

Theoretical frameworks … which emphasise the simultaneous significance of global, national and local forces on the development of higher education offer a powerful conceptual frame. However, while this provides an understanding of the relationships between systems of higher education and globalization, it does not explicitly address the role of higher education in development (Naidoo, 2010, p. 81).

Marginson and Rhoades (2002) were taken by the new freedoms and potentials of the global setting. The perspective of the glonacal paper is an exuberant ‘outwardlookingness’, ‘a positivity and aliveness to the world beyond one’s own turf’ (Massey, 2005, p. 15). Like others (e.g. Beck, 2000), they imagined that world in non-neoliberal and cosmopolitan terms. However, the paper's examples of positive mixing were largely drawn from student mobility into the Anglophone world, and though there were passing references to resource inequalities, brain drain and English language bias, in the paper, Euro-American centrism and monopoly of global knowledge seemed to be secondary concerns. The multi-scalar glonacal heuristic can be used in a critical analysis of hegemonic power in higher education (e.g. Marginson, 2022d), but this was not done in 2002. The glonacal paper also missed the implications of the vacuum in global governance in higher education and the lack of momentum for the global common good. Freedom from direct political regulation by nations is attractive but has its downsides. This has implications for mobile students who are not adequately protected.

### Did the glonacal paper get it right?

The glonacal paper highlighted the expanding activity in the global scale, dismissed claims that globalization was undermining the nation-state in higher education and emphasized higher education’s autonomy as a sector, its distinctive historical trajectory within the larger context. It distanced itself from the idea of higher education as a branch of the global knowledge economy. How well have these judgments travelled over the 20 years?

Since 2002, the spatial dynamics of the context have changed in important ways. The momentum for the globalization of capitalist economies has slowed. The growth of cross-border trade has levelled off, and protectionist practices have increased. There is a partial retreat from global supply chains and offshoring. The economic role of multinationals has slightly declined (The Economist, 2022). There is more strident opposition to open borders and cosmopolitanism (Rizvi et al., 2022). Nation-bound thinking is rife, and the once widespread idea of an Americanized globalization undermining the nation-state has now vanished. It is no longer necessary to put the nation-state back in the picture as it was in 2002.

At the same time, the trajectory of higher education and science has been partly decoupled from the political economy, as the glonacal paper suggested. While higher education is affected by geo-political tensions (e.g. technological competition between the U.S. and China), and from time to time there are blockages in student mobility and to a lesser extent in the flow of messages and knowledge, there is no sign of a general retreat from cross-border activity in higher education and knowledge akin to that in manufacturing, trade and politics. On the contrary, in higher education, there has been continuing strong growth in global activity, without diminishing the nation-state, again as stated in 2002.

Global convergence and integration have three modes: global connections, global diffusion of models and practices and global systems (Marginson, 2022d). In all three modes, the globalization of higher education and knowledge continues to markedly advance.

Global connections include the cross-border movement of students, researchers and other personnel, university agreements and consortia (Beerkens, 2004), twinned degrees and similar arrangements, online programmes, and research partnerships and publishing. Despite ebbs and flows in student mobility into particular countries, it grew from 1.9 million students worldwide in 1998 to 6.1 million in 2019 just prior to the COVID-19 pandemic, and it is likely that the growth pattern has resumed in the post-pandemic period. Comprehensive data on researcher and faculty mobility are lacking, but it is likely the annual growth rate exceeds that for first-degree students. In 2019, 22% of all doctoral students in OECD countries were non-citizens (OECD, 2021, pp. 212–223). Global diffusion of information, ideas, models and behaviours has been quickened by intensified global communications (Roberston et al., 2023) and the ‘discourses of the global’ (Massey, 2004, p. 10) they carry. There is partial convergence around Euro-American norms of the university in management, epistemic structures, academic ranks, the doctorate, certification and the degree ceremony, though this commonality should not be overstated, and non-university institutions are more diverse. There is extensive policy borrowing in relation to financing systems and incentives, administrative technologies such as quality assurance and research assessment, and institutional performance management, social engagement and public accountability, though a more conclusive comparison of trends requires detailed empirical study.

Global systems include the communication and information networks in higher education, global science, and global comparisons, classifications and rankings. These systems are partly detached from nation-states, while influencing nations and institutions from outside. It would be hard to overestimate the impact of communications in integrating higher education and research. Global science output has grown by more than 5% per annum since 2000, and almost one-quarter of all published papers now have international co-authors. More than 60 countries have achieved a level of output that suggests their own doctoral training capacity, double the number of two decades ago (Marginson, 2022c). Global comparisons of higher education, including both university rankings and the OECD’s annual data set on national systems, *Education at a Glance*, have become entrenched in policies, institutional strategies and practices and the decisions of faculty, students and their families, donors and employers (Hazelkorn & Mihut, 2021).

What about the position of nation-states vis a vis the global? Arguably, between the mid-1990s and mid-2000s, the globalization of communications and the dissemination of policy ideas and institutional modes were transformative. They triggered a process of disembedding and re-embedding of national higher education systems (Beerkens, 2004). When the dust settled, systems and institutions found themselves newly relativized within the global scale—by rankings, by the reform agendas of the EU and multilateral organizations, by the plethora of non-government organizations in the global education space (Hazelkorn, 2020), by the expanding science networks, and by a shared teleology that all international connections were desirable (the last may now be faltering). However, politics and policy were and remain national and international, but not global. Appadurai (2014) notes this is especially the case in the ‘global South’, where inner political life is dominated by nation-building rather than global issues (pp. 487–488). Compared to 2002, states have a diminished capacity to control information and imagining in higher education (Rizvi, 2011, p. 183). However, the structural materiality of the nation-state remains crucial to the sector, setting funding and legal forms, affecting cross-border flows of people, and in some countries, though not all, influencing cross-border research collaboration. At the same time, to a varying extent, nation-states have endorsed and assisted global convergence in higher education. Despite cultural tensions, few nations have actively refused it.

In summary, in higher education, the nation-state has been relativized but not diminished by the growing activity in the global scale. By adapting to the ‘global competition state’ (Cerny, 1997) and framing higher education for a global knowledge economy, nation-states have strengthened their agency. The simultaneous advance of national agency and global relations not only suggests that they are mutually constituted (Bayly, 2004), it is a clear illustration of the open multi-scalar vision in the 2002 glonacal paper. However, the glonacal paper underestimated the extent to which many governments would buy into a marketized imaginary of higher education in the knowledge economy. While this has not transformed (and cannot transform) higher education into a wholly capitalist sector, and prestige continues to be more important than revenues in driving competition (Marginson, 2013), the market imaginary has left its mark in local, national and global practices.

With this caveat, the core theory and predictive judgments in the glonacal paper have stood up well. The authors have not had to continually revise the definitions and meanings of glonacal, in contrast with the changing definition of ‘internationalization’ (Knight, 2003). But did the paper remake comparative higher education research as it hoped?

Yes and no. It helped to bring spatiality to higher education studies. However, some see ‘glonacal’ merely as a synonym for ‘global’, with the multi-scalar idea a bridge too far. In a summary of literature on international and global higher education, Lee and Stensaker (2021) note three differing propositions: the role of nation-states is declining; the nation-state remains important; and institutions adapt to global norms (which could be compatible with each of the other two). This indicates that within higher education studies, there is still little clarity on a central spatial issue. Zero-sum thinking clouds judgment—the assumption that global norms and activities *must* reduce the authority, role or effects of nation-states. This in turn indicates the continuing influence of methodological nationalism (see below) and consequently, a widespread inability to grasp the multi-scalar character of the sector.

The standard nation-bound understanding of higher education is still largely intact despite the growth of global science and two decades of research on cross-border higher education. Comparative education studies are mostly still nationally bordered, though some draw on the glonacal heuristic (e.g. Kosmützky, 2015). The glonacal idea is influential but not dominant. Ironically, this has helped to maintain its critical edge. Pitched against the orthodox nation-bound reading of higher education, the glonacal idea still has something new to say. There are also gains to be made by taking it further. This seam is not exhausted.

## Making space in higher education

Space–time is one of the social-material coordinates of higher education. It intersects with other primary coordinates such as capital and class, political culture and regulation, language and knowledge, and hierarchies of ethnicity-race, gender and sexuality. Space is emerging and constructed. Space-making combines materiality, imagining and social practices. This section reviews space in general and in higher education, the multiplicity of space and the scope for agency, and tendencies to difference/sameness and openness/closure.

### Space as relational and multiple

The conclusions of Marginson and Rhoades (2002) about multi-scalar higher education, which originated in raw observation, paralleled literature about space and scale in human geography (e.g. Herod, 2008; Lefebvre, 1991; Marston et al., 2005; Watkins, 2015) and global history (e.g. Conrad, 2016). Multi-scalar analyses are common in social science, including political science, economics and scientometrics using bibliometric data (e.g. Hennemann et al., 2012) and studies of innovation (e.g. Guan et al., 2015; Gupta et al., 2007). Human geography is helpful for the present paper, including the work of Doreen Massey as in *For Space* (2005).

Social spaces take many forms, such as markets, networks, cities, villages, multi-site organizations and geo-cognitive scales like the global, national, regional and local. Social spaces are not pre-given structures lined up and waiting to be populated like a row of empty aircraft hangers. Harvey (2005) refers to ‘an actively produced field of spatial ordering that changes sometimes quickly and sometimes glacially over time’ (p. 244). Space is not a pre-existing blank sheet written on by people and events. Space emerges through the actions of people, and their social practices and connections—for individuality and sociability are inseparable (Massey, 2005, p. 58)—which become combined with non-human elements and coordinates. Space is all of this together, and each space is continually being created. Spaces are moving, evolving constellations of social-material relations that humans make for themselves.

For Massey, space and time are heterogeneous and intersect. ‘If time unfolds as change then space unfolds as interaction’ (Massey, 2005, p. 61). Time means the history of agents: it can be understood as ‘narrative’ and especially as ‘trajectory’ or life journey. Space is where the multiple agentic trajectories intersect. ‘Space is the *social* dimension … in the sense of engagement within a multiplicity’ (p. 61, emphasis in original). In spaces, human agents encounter ‘coeval’ (coexistent) others with their own distinctive trajectories, in a ‘meeting up of histories’ (p. 4). There are also gaps, missed intersections. Space is ‘the sphere of relations, negotiations, practices of engagement, power in all its forms’. ‘Space is the dimension which poses the question of the social, and thus of the political’ (Massey, 2005, p. 99). Active agents are integral to space making, though space is constructed by past as well as present agents, and resources with which to exercise agency and fashion social spaces are unequally distributed.

Massey (2005) sharply critiques those imaginings in which space is abstract and place is concrete (p. 183). Her specific target is the influential global/local binary. In this mode of thinking, global forces are seen as external, economic and dynamic while ‘local place’ is seen as internal, organic and fixed-residual, the victim of globalization, doomed to be subsumed by or defended from the global. Massey, who is well aware of the power of global capitalism, debunks ideas of globalization as an abstract universal force and the local as prior to social practice. Space and local place are equally dynamic, social and constructed by agents, ‘an open ongoing production’ (p. 55). ‘Position, location, is the minimal order of differentiation of elements in the multiplicity that is co-formed with space’ (p. 53). Global activities ‘are utterly everyday and grounded, at the same time as they may, when linked together, go around the world’ (p. 7). As Larsen and Beech (2014) state, ‘the global is not just some space out there, without material basis. It is produced in local settings’ (p. 200). But locations are not all equivalent, and the different local agents have unequal capabilities to act. ‘Put bluntly, there is far more purchase in some places than others on the levers of globalization’ (Massey, 2004, p. 11). Her example is London. Likewise, the leading Anglo-American universities dominate existing global practices in higher education. Not all universities exercise an equivalent role.

Space for Massey is unfinished, always becoming. It continually combines previously unconnected trajectories (Massey, 2005, pp. 39, 41, 59). There is always movement. ‘We are functioning in a world that is fundamentally characterized by objects in motion’, states Appadurai (1999). It is also ‘a world of structures, organisations and other stable social forms. But the apparent stabilities that we see are, under close examination, usually our devices for handling objects that are characterized by motion’ (p. 230). There is also unpredictability and contingency. ‘There are always loose ends’ (Massey, 2003, p. 5).

Massey wants to ‘uproot “space”’ from concepts such as fixture, stasis and closure and ‘settle it’ among relationality and heterogeneity’ (Massey, 2005, p. 13), allowing the unknown to appear, ‘the positive creation of the new’ (p. 54). Multiplicity, in all its forms, ‘diversity, subordination, conflicting interests’ (Massey, 2005, p. 61) is likewise foundational. Space is the sphere of ‘coexisting heterogeneity’ (p. 9), ‘of the possibility of the existence of plurality, of the co-existence of difference’ (Massey, 2003, p. 3). The difference is neither static nor discrete but continuously co-evolving, fusing and emerging (Massey, 2005, p. 21).

### Agents as space makers

Multiplicity is inherent in space-making in higher education because agents in all forms—individual persons, groups and networks, institutions and other organisations, governments and their agencies—have autonomous trajectories grounded in their own self-awareness.

From a social realist perspective in sociology, Archer (1995, 2000) expands on the irreducible autonomy of human agency. For Archer, agency and structure are different aspects of a stratified social reality. Each is not fixed but is evolving, emergent. They intersect and affect each other, but they are also autonomous and heterogenous. Archer contrasts this position with those of Giddens and Bourdieu. In Giddens’s (1986) theory of structuration, structure and agency are constituted on a reciprocal basis, implying that ultimately they form a single identity. For Bourdieu (1984), society is installed in each person through the habitus, socialised norms or tendencies that guide thought and behaviour. Agency struggles to find a path through the structural determination. Archer (1995) agrees that structure is prior to agency. ‘Structure’ includes material resources; ‘ideational’ culture, including language, knowledge and information; social relations; and configurations of power. ‘Society takes shape from, and is formed by, agents, originating from the intended and unintended consequences of their activities’ (Archer, 1995, p. 5). For example in higher education and knowledge, strong agents—leading universities and scientists, Anglophone countries and parts of Western Europe—confront other agents with determining structural force. But no structural determination can be absolute. ‘People are not puppets of structures because they have their own emergent properties’ (Archer, 1995, p. 71).

‘People are capable of resisting, repudiating, suspending or circumventing structural and cultural tendencies, in ways which are unpredictable because of their creative powers as human beings’ (Archer, 1995, p. 195). They have independent potentials because of their capacity for reflective consciousness, their ‘inner conversation’ (Archer, 2005). This emerges early in life and is universal. It develops out of both biological constitution and interactions with the external environment (Archer, 2000, p. 50). It enables people to reflect on their social context and act reflexively towards it, individually or collectively (p. 308). Agential powers are conditioned by context but not wholly determined by it (pp. 10, 269). Different agents can have varied responses to common external conditions (p. 70). The notorious individuality of academics, the herding cats syndrome is, in a sense, the human condition.

People, institutions and nations in higher education sometimes but not always break new ground in making space, as well as when they populate and use space. For example, consider what was once the startling innovation of the Global Schoolhouse in Singapore (Sidhu, 2005). When that strategy emerged nothing like that kind of space making in education had been seen before. People and organisations in higher education are both constructed and free and also aware of both (Archer, 1995, p. 2).

### Materiality, imagination and practice

**Fig. 1:**
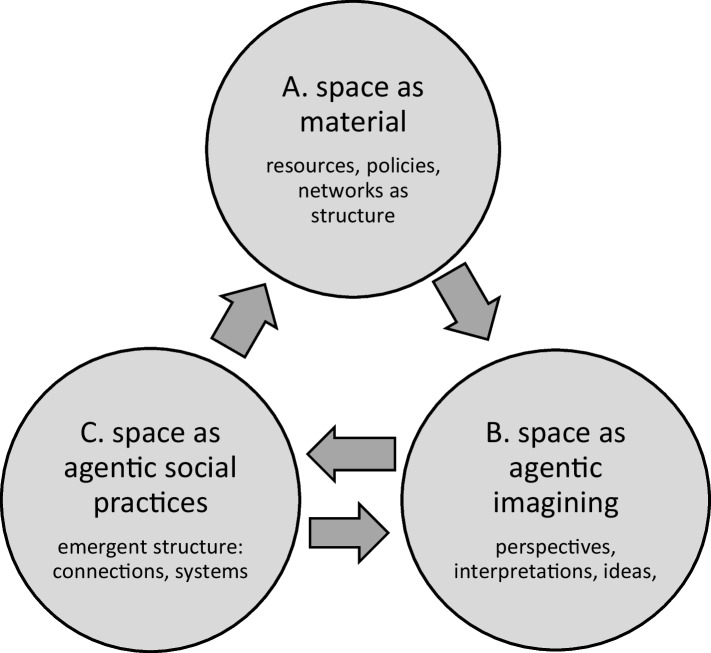
Geo-cognitive space in higher education as materiality, imagining and social practices

Henri Lefebvre’s *The Production of Space* (1991) suggests a three-way relation between space as material, space as imaginative and space as social practices and social relations (e.g. pp. 11, 27). Anderson (2006) labels the constructed spaces called ‘nations’ as ‘imagined communities’, highlighting both the imagination and social formation. The three elements continually interact. The challenge for social science, including higher education studies, is to move from the suggestive and fluid theorizations of Lefebvre, Massey and others to concepts that are operationalizable as empirical observations. Figure 1 models a version of Lefebvre’s three-way schema for scale as space in higher education (for an antecedent parallel of Fig. 1 in geography see Cox, 1981, p. 136). The model could be used for investigating the global scale (e.g. as in Marginson, 2022d) or the national, local, regional or city scales. Like all such models, Fig. 1 fixes and simplifies an irreducibly complex and continually moving reality.

In Fig. 1, the material domain A includes pre-given structures like communication networks, inherited institutions, infrastructures, language of use, laws, policies and the whole apparatus of economic resources including sunk investments and ongoing funding. The lower two domains B and C especially embody individual, group and organizational agency, as on one hand imaginings and discourses and on the other hand the practical work of education, research and service in higher education, including co-production with social stakeholders. The three domains A, B, and C closely interface. Various theorizations have focused on the overlaps. For example, Lefebvre’s ‘spatial practice’ embodies perception and interpretation. ‘Spaces of representation’ include space as lived and felt (Larsen & Beech 2014, p. 200). For Massey (2004), identity is ‘both material and discursive’ (p. 5). For James and Steger (2016), globalization combines practice and consciousness. Appadurai’s scapes interpolate subjective ‘perspective’, whereby agents envision global cultural flows, into an otherwise impersonal notion of economic globalization (Appadurai, 2014, pp. 483–484).

Imagination and discourse in domain B are key elements in space-making. ‘Changing the metaphors we use to describe the world does not change the way the world actually is, but it does change the ways we engage with the world’ (Herod, 2008, p. 226). Watkins (2015, p. 508) refers to three kinds of collective spatial imaginaries: imaginaries of places; idealized spaces, such as a harmonious nation, or a cosmopolitan world of global citizens; and ‘spatial transformations’, such as discourses of internationalization, or higher education as a global competition in the knowledge economy. James and Steger (2016) distinguish four levels of lived meaning, which they note have been carriers of globalization, with successively deeper resonance in society: (1) ideas; (2) ideologies; (3) social imaginaries (Taylor, 2003), which frame the shared understanding of an age, such as neoliberalism; and (4) ontologies, which constitute a shared sense of reality (James & Steger, 2016, pp. 25–26). Spatial imagining in domain B is conditioned but not confined by pre-given materiality in domain A.

In domain C, agents attach interpretations in domain B to material resources from domain A to build activities, programmes and organisations, including social relations inside and beyond higher education: manifestations of spatiality ‘necessarily embedded material practices which have to be carried out’ (Massey, 2005, p. 9). Agency becomes embedded in a new structure. For example, in domain B governments conceive science as a global arms race in innovation or as integral to nation-building, as in emerging science countries (Chinchilla-Rodríguez et al., 2019). On the basis of prior facilities in domain A, they fund expanded national science capacity in domain C. This in turn augments personnel and infrastructure in domain A and stimulates parallel science-building cycles in other nations (Marginson, 2022b).

The flow of arrows around Fig. 1 is significant. At the same time, the agency-heavy domains, imagining/interpretation (B) and social practice and relations (C) continually constitute each other. While there is a sequential relation between imagining in domain B and social practices in domain C, practical experience in domain C also suggests possibilities and limits for reflexivity in domain B. For example, in the late 1990s, in domain C stand-alone ‘e-universities’ failed to attract student customers to the online-only format. This triggered reflection in domain B, among the institutions, corporations and governments that had sponsored the unsuccessful online universities. Later content-rich MOOCs were conceived in domain B and implemented in domain C, through existing institutions, first of all Stanford University. This model proved much more attractive to students.

The relation between imagining in domain B and social practices in domain C is never automatic. The same imagining of space can be associated with a wide range of practices. Take the cosmopolitan idea of a world of inter-connected global citizens (domain B). There are multiple themes and strands in domain C: programmes focused on openness, diversity (Rizvi, 2011), broad inclusion and tolerance; the argument of Santos (2007) for epistemic inclusion in an ‘ecology of knowledges’; the use of cross-cultural mixing, multiplicity and cultural hybridity as learning tools (e.g. Rizvi, 2005); and institutions that build cross-cultural ‘competences’ in personnel to augment their own spatial freedoms.

League table rankings were first conceived in domain B by scholars in Shanghai and journalists in London, drawing on norms of scientific production and economic competition respectively. This simulated a worldwide–higher education sector in the form of a global market of ‘World-Class Universities’ (domain C). The simulation became real, guiding investment and strategy, becoming reproduced in domain A with structural force as organizational priorities and resource allocations. Across the world universities and countries became locked into incentives and models that they would never have chosen for themselves. For example, in sub-Saharan Africa, global rankings can hardly be ignored but are felt as coercive, pressuring institutions ‘to do things not necessarily within the realm of burning institutional needs’ (Teferra, 2019). Global rankings are a striking example of the potentials of spatial imagining when institutionalized in a successful practical prototype. This also shows that spatial imagining can be reductive as well as productive. Global market competition can diminish mutuality and cooperation.^3^ ‘Social imaginaries circumscribe what is deemed possible or legitimate to think, act and know’ (Stein, 2017, p. 329).

The above examples refer largely to global space-making in higher education, but Fig. 1 can also model the construction of social space in local higher education institutions, and in nations or regions. In national higher education, government works strenuously to conceive, implement and ideologize social practices in domains B and C, continually reshaping the material infrastructures in domain A that make visible the national system space. Domain A is demarcated and calibrated by policies, priorities, hierarchical orders and above all, by budgets. In the pan-national regional scale, in the last 25 years, countries in the European Union have built a European Higher Education Area and European Research Area from the ground up, drawing on a domain B vision of a shared future and growing integration over time. Spatial relations have been very deliberately fostered: for example research funding in domain C requires collaborative cross-country teams and that gives form to the European Research Area as a shared and single space. More slowly and less completely, the Association of Southeast Asian Nations is likewise constituting a regional space in higher education (Robertson et al. 2016).

### Openness/closure and the strategies of agents

Space making is ‘a shared historical process that differentiates the world as it connects it’ (Massey, 2005, p. 139). It entails combined and uneven development. In part inequalities between agents can be understood in spatial terms. Marginson and Rhoades (2002) suggested the notion of ‘spheres of agency’, meaning ‘the parts of the world’ reached by an institution, a unit or a national higher education system, its ‘webs of activity and influence’ (p. 293). All agents in higher education have limits, not just in their history, inherited resources and language of use, but positioning, and scope for re-imagining. The limits are constantly changing, as the strategies and trajectories of agents intersect with the unfolding dynamics of mutually constituted space, re-forming opportunity and hierarchy. Continuing fluctuations of openness/closure and homogenization/heterogenization (sameness and diversification) are both engineered by agents and the uncontrolled outcome of their interactions, with implications for multiplicity in all of ‘diversity, subordination, conflicting interests’ (Massey, 2005, p. 61). These movements combine to produce a complex, shifting assemblage, and play into – and are instruments of—the ever-evolving relations of power.

In his account of the political economy of expanding communicative networks, Castells (2000) explains how ‘metropolitan concentration and global networking … proceed simultaneously’ (p. 225). Networks are both open and dynamic, extending to every possible node while intensifying the links between existing nodes. As nodes (such as the best positioned and resourced universities, cities, and national systems in higher education) accumulate more connections they attract greater resources and build capability as agents. Meanwhile expansion enables new nodes to form and strengthen. There is no essential conflict between network inclusion and concentration. They can be positive-sum. However, agents also use selective strategies of both openness and closure to secure advantage.

For long global science was dominated by US scientists, underpinned by national science funding and the concentrated resources and inherited legitimacy of the leading universities. Both grass-roots American science and national policy favoured free global expansion of collaborative science on the basis of open networked cooperation. The policies favouring cross-national collaboration inside Europe helped regional science, including the smaller national science infrastructures in the South and East, to accumulate national and regional weight and build global impact. The EU countries supported free networked association outside Europe, including partnerships, while region-building at the same time. Up to the early 2000s, global science functioned largely as a Euro-American duopoly with outliers in countries such as Japan, Israel and Australia. North America was the leading component of the duopoly. Euro-American norms and institutions, and the English language, regulated inclusion: both epistemic content and professional norms were closed to other cultures and languages, including endogenous knowledge from the global South (Marginson & Xu, forthcoming). The resulting global science space confronted other agents as a fixed structure, encouraging universal participation in a closed agenda. Both the openness and the closure facilitated Euro-American and especially US global leadership.

‘What is at issue is the nature of the relations of interconnection – the map of power of opennesss’ (Massey, 2005, p. 171). Strategies of closure, like concentration, can build agency; and sometimes—not always—closure is used to partition space so as to block activity by other agents. What matters is not the abstract spatial form in itself but ‘the social relations through which the spaces, and that openness and closure, are conducted’ (p. 166).

Because all space is multiple and relational, no closure is ever complete. Over time every space ‘escapes in part from those who make use of it’ (Lefebvre, 1991, p. 26). The terrain is continually shifting and the strategic logics change with it. Between 1985 and 2020 Chinese scientists made such effective use of networks in US-led global science, especially through bilateral collaboration and co-publication between scientists in China and the US (Lee & Haupt 2020) and through benchmarking with Euro-American universities, that the total volume of China’s global science output came to surpass the US level, and the leading Chinese universities achieved parity with their US counterparts in high citation papers in the physical sciences and related disciplines. At the same time the open networking regime in science facilitated the emergence of a more multi-polar higher education world, with rising non-Western middle powers in science including India, South Korea, Iran, Brazil (Marginson, 2022a, 2022c). The global science space – perhaps the global higher education space—partly ‘escaped’ from Euro-American domination. The rise of China, increasingly perceived by the US government as a strategic rival in technology, triggered a change in the US government’s prima facie support for open international collaboration. In 2018 the US’s China Initiative drew a tight national boundary that criminalized or stigmatized some plural China-US activities (Lee & Li, 2022) and reduced overall China-US collaboration. Certain Chinese doctoral students received more restricted visas while others could not enter the US. ‘The closed geographical imagination of openness, just as much as that of closure, is itself irretrievably unstable’ (Massey, 2005, p. 175).

#### Sameness and difference in space making

Such changing moves feed the ongoing oscillations between homogenization and heterogenization. With globalization ‘many incongruous facets of human existence have been forced together into a giant tumbler’ (Yang, 2019, p. 68, citing Odora Hoppers, 2009). On one hand difference is flattened: activity is pressed into common systems, as in global publishing science and global rankings. On the other hand there is also greater visibility and potency of difference when the various cultural, scholarly and institutional traditions are brought closer together. The same models and ideas in higher education are ever more widely spread yet they undergo diverse reworking.

For Appadurai (1990) the relation between cultural homogenization and cultural heterogenization is ‘the central problem of today’s global interactions’ (p. 295). Pieterse (2020) sees differentiation and universalism as twin ‘drivers’ of human affairs (p. 235), successively giving way to each other. Similarly, perhaps, Cerny (1997) finds that ‘global competition states’ follow differing strategies of ‘adaptation and transformation’. By turns they imitate each other to maintain parity, and innovate to secure advantage, in a continuing ‘dialectic of divergence and convergence’ (p. 265). Harvey (1990) notes that the more unified is the space, ‘the more important the qualities of fragmentation become for social identity and action’ (p. 271). For Marston et al. (2005) ‘complex systems generate both systematic orderings and open, creative events’, with orderings being the more common occurence. Variations often cluster and become mimetic over time (p. 424).

Arguably, however, homogeneity and heterogeneity are not symmetrical or necessarily always zero-sum, and the oscillating double movements between the two are not a dialectic. If agentic difference is constantly emerging the last word must be with heterogeneity. ‘If space is genuinely the sphere of multiplicity’ with diverse trajectories ‘there will be multiplicities too of imaginations, theorizations, understandings, meanings’ (Massey, 2005, p. 89). Hence the multiple modernities and varieties of capitalism in heterodox economics and sociology, which contradict ‘the universality and homogeneity characteristic of conventional social theory’ (Beck & Grande, 2010, p. 413). This suggests that as capacity in higher education expands and becomes more distributed worldwide, there will be greater diversity between systems, possibly even while the shared architecture is being strengthened.

The literature on higher education exhibits differing positions on the relations between homogenization and heterogenization. Institutional theory emphasizes homogenization. It models a uniform spatial structure with deductive forms in which ‘world society’ in the global scale programmes the content of what are seen in the theory as the key agents, institutions. The university is a hinge between global and national/local scales and as such a carrier of cultural ‘universalization’ (Frank & Meyer, 2007, p. 287). Universities follow liberal Americanized ‘global scripts’ (Schofer & Meyer, 2005) and also imitate each other. Arguably this theorization downplays national variation, and local agentic initiative. Perhaps, more generally, isomorphism in common environments is overplayed in higher education studies (Valimaa & Nokkala, 2014).

At the level of persons and disciplinary groups, agents are ‘contextual’ (related to context) but not necessarily ‘contextualized’ (embedded in context) (Xu, 2022, p. 134). They have room to move. At the level of national systems, consider the never-ending variations, within the common worldwide setting, in the handling, adoption, modification and refusal of neoliberal policy agendas. In a comparative overview Cummings concludes: ‘The classical debate focuses on the extent to which educational systems become more similar or retain distinctive structural differences over the course of modernization and globalization… the evidence is overwhelmingly in favour of the differences position’ (Cummings, 2006, p. 2).

Institutions can be locked into national systems and in that respect more confined than are persons or the nations themselves. Where there is institutional autonomy in mission and strategy it often seems to be under-utilized. Still, from time to time institutions break the mimetic mould. Friedman (2018a) notes UK and US universities with parallel resources and status but distinctive internationalization strategies (pp. 131–132). In NYU’s highly original global degree students study for at least one year in two of New York, Abu Dhabi and Shanghai. In the ‘fluidity, indeterminacy and open-endedness’ (Pieterse, 1995, p. 46) of the global scale, many institutions pursue actions that are only semi-regulated. Their imagined global spaces overlap (Calhoun, 2003) and each extension of the shared spatial horizon expands the collective sources of the organizational self.

## Scale and higher education

This section discusses geo-cognitive scale as one kind of constructed space in higher education. It expands on the multiple scales, imagined and practised, and on scalar heterogeneity and primacy, cross-scalar movement, and the national/global coupling.

### Multiple scales

There are many definitions of geo-cognitive scale (for a fuller discussion see Sheppard & McMaster, 2004). Simplifying, scales are recognized geographical meta-spaces that vary on the basis of scope and proximity. ‘Scale is a produced societal metric that differentiates space’. The ‘social ownership’ of scales is ‘broad-based’ (Marston & Smith, 2001, p. 615).

**Fig. 2:**
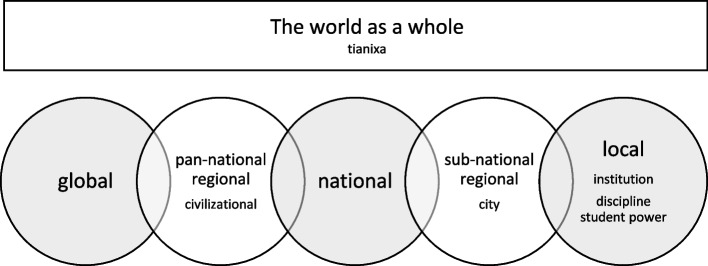
Geo-cognitive scales in higher education

Conscious scales in higher education (see Fig. 2) include the *world as a whole* and everything in it, including all the other scales; the *global* scale of relations at the planetary level; the *pan-national regional* scale, as in the European Union; the *nation*; the *sub-national region* such as state or province, and the *city*, a scale often important in higher education (Goddard & Vallance, 2013; Soja, 2010); and the proximate *local scale*. The glonacal paper’s configuration of global, national and local radically simplified these six scales. Arguably the three glonacal scales are primary in higher education, though as noted, the local is more than just an institutional scale, and the region is a primary scale in Europe.

The first of the scales, the single ‘interrelated, interconnected and interdependent’ world as a whole (Rizvi, 2006) is surprisingly under-developed for space making in higher education. The ancient Chinese idea of *tianxia*, a world without borders held together by ethical relations rather than coercive practices, is one starting point for such imagining, provided *tianxia* is understood as world-centred rather than China-centred (Yang et al., 2022). Robertson et al. (2023) add ‘*civilizational states*’ (p. 5) to the list of scales. To adequately understand China, and perhaps the US, requires a scalar concept with a cultural reach that is larger than the nation-state but distinct from the territorial conquest form of imperialism.

The distinction between the national and global scales leads to the identification of two different kinds of cross-border relations. On one hand there are ‘inter-national’ relations between nations, or between organisations or other agents located in two or more nations. On the other hand, there are ‘global’ relations that pass over nations and integrate agents at planetary level.

‘Scaled social processes perpetuate specific productions of space’ (Marston & Smith, 2001, p. 616) but the extent to which a scale is practised is an empirical question. Scales are ‘geo-cognitive’ because like all space they combine continually emerging material elements, and thought and discourse, with social practices of space (Fig. 1). Contact and connections alone are not sufficient to constitute scales, which entail conscious imagining and transformative social relations (James & Steger, 2016, p. 22). Imagining scale is crucial in bringing it into being. For example, James and Steger note that the ‘global’ has become especially linked with two imaginaries, ‘the market’, which is the most readily recognized association, and the ecological idea of a world in which everything is connected (p. 28). Scalar imagining and practices in turn institutionalize what agents do, reproducing the scales in apparently stable ways. People think globally, act locally, feel national, see as a state, and so on.

Are scalar distinctions then nothing more than agentic imaginings? Some geographers answer 'yes' to that question, limiting scale to methodological and epistemological status rather than ontological status. For Jones et al. (2007) scales do not exist and because scale thinking can impose a misleading hierarchy, it is better to read social practices in terms of a ‘flat ontology’. Against this it can be argued that scales, like other human-made spaces, *do* exist in that they shape social relations and generate material manifestations. For example, the nation is an ‘imagined community’ and also one that is practised. Its agents define its territory and enforce that claim by coercive means and engineered hegemonic consent. The nation-state confronts higher education institutions and other agents with the awesome structural force of laws, regulations, customs, language, economic management, financing, policies, programmes and the like. At that point, despite the fictional origin of the nation and the arbitrary and ambiguous character of its territorial borders (Vaughan-Williams, 2008), it is difficult to deny that the nation and the national scale exist! On the contrary, the nation form has become so pervasive as to be taken for granted. This tends to conceal the continuous and strenuous work entailed in the construction of nations, including the formation and propagation of their ideologies and narratives.

There is no bedrock essential scale, the true unchanging site of identity. The most proximate local scale is the self-regulated domain of daily life and neighbourhood, and in higher education, the place of work and study. Though there is a material ‘immanence’ in the local (Woodward et al., 2012, p. 204) it is no more fixed than other scales. Like the nation, ‘locality … has always had to be produced, maintained and nurtured deliberately … the local is not a fact but a project’ (Appadurai, 1999, p. 236). In the global scale multilateral geopolitics and global capital pre-structure relations only up to a point. Openness and mobility enable fecund conditions. Diasporic communities use travel and media (Appadurai, 1996, 1999), finding hybridized spaces between nations that blur the inherited geopolitical, socio-economic or ethnic-racial hierarchies (Pieterse, 1995, p. 56). For Brooks (2018) international student mobility itself constitutes a distinctive space ‘of identification and belonging’ (p. 2).

Agentic perceptions, potentials and experiences of scale vary on the basis of resource and position. In higher education and knowledge the formation of global relations has been historically dominated by agents in the Euro-American countries. Yet as noted, a more multi-polar higher education world is emerging. Here global communications have facilitated the evolution of a more distributed science capacity at local and national levels. The map of ‘World-Class Universities’ is more diverse by nation (Marginson, 2022a, 2022c). Emerging country systems quicken their development by accessing the global pool of knowledge and network freely with each other without being blocked by strong countries. The concentration of network power has diminished over time (Wagner et al., 2015).

Scalar activities are also differentiated within nations. Friedman (2018a) reviews the ‘creation, legitimation and differentiation of cosmopolitan capital among different groups of students’ through ‘global citizenship’ programmes in contrasting UK universities. ‘Cosmopolitan capital’ refers to knowledge, skills, attributes and dispositions that confer global advantage. The content of that capital varied on the basis of institutional position in a national hierarchical order. Friedman’s ‘Old Elite’ university was neo-imperial. It saw its faculty and graduates as natural global leaders. There was no need to foster cosmopolitan capital. ‘New Elite’ university focused ‘conspicuously’ on transforming itself into a ‘global university’ (p. 6), redefining its identity. It prepared its students for globally mobile work and global civil leadership. ‘Urban Access’ university focused on recruiting international students and discussed the benefits of culturally diverse student life, but its larger priority was local employment of local students not the global scale.^4^ ‘Valley Access’ university recruited international students for revenue but gestured only nominally towards global education.

In these examples the varied positioning of higher education in two local scales – those of the institution, and its contiguous community – articulated with a global scale differentially practised. Each scale ‘is also a product of relations which spread out way beyond it’ (Massey, 2004, p. 6). In the multi-scalar setting the scales overlap and co-penetrate, as the glonacal paper stated. Interviewees in Friedman’s (2018a) ‘Old Elite’ university know that their local activities construct global models and knowledge in higher education. The reverse is also true: those same global models constitute local activities, albeit in other places. The more that multiple scales intersect, the more that practices in each scale are opened to change, though in such intersections the dominant agents are the least likely to change themselves.

Multi-scalar spatiality also leads to ambiguities. In systems where institutions are closely embedded in the state, the boundary between national and local-institutional scales can be unclear. In the global scale, the extent of convergence and integration (Pieterse, 2020, p. 234), and the boundary between ‘connection’ and ‘integration’ (Beerkens, 2004, p. 16) are again unclear and the subject of conflicting perspectives and claims. Is the connection ‘inter-national’ or ‘global’? International relations presume that the respective nations are unchanged. Global relations can be more disruptive, punching holes in the national border.

### Heterogeneity of scales

As noted, geo-cognitive scales in higher education are heterogeneous—associated not only with differing scope and proximity but also with various experiences, perspectives, configurations of governance and power, and boundaries of the possible. Multiplicity derives not just from Massey’s coeval agents with distinctive consciousness and trajectories, but from this larger diversity of scales as spaces. Cross-scale relations are a potentially open division of labour between differing social constellations that taken together expand the potentials of higher education. Because the scales are not equivalent, scale-based causation in higher education is more than random. For example, the nation-state has an often decisive role in political matters, while communications are quintessentially global in character, and because of this, knowledge is especially global. Like language, knowledge is also grounded in local collectivity.

Table 1 considers and compares the national and global science systems. The systems are very different. There are also shared objects that appear in both systems.

**Table 1:** Distinctions and relations between global science system and national science systems

	Global science system	National science systems
Space as material		
Core material components of science system	Knowledge, scientists, networked communications, professional organisations, norms and practices	Knowledge, scientists and norms, academies, universities, government agencies, laws, policies, budgets, infrastructure and equipment
Enabling material conditions of science system	Communications infrastructure, plus resources, institutions, policies and rules, mostly provided by nation-states	Economic resources, political stability with policy commitment to scientific activity and maintenance of institutions
Knowledge contents of science in system	Papers admitted by Web of Science and Scopus, i.e. global bibliometrics, nearly all in English	Much of the global papers plus further nationally produced outputs, including (where relevant) non-English works
Space as agentic imaginings		
Normative centre of science system	No normative centre but network leadership often largely concentrated in science strong countries	Nation-state: the interests of no agent outside the nation can ever take priority over the bounded national interest
Imagined boundary of science system	World society	Jurisdictional border of the nation-state
Imagined community in science	Disciplinary networks of scientists, professionally competitive but bound by collective knowledge and ethos	Nation and its scientific institutions, embedded in innovation-oriented industrial economy and society
Imagined mission	Scientific discovery, working together (often across borders) to address global challenges and problems	National security and competitiveness (including innovation) in global setting, national well-being and prosperity
Space as agentic social practices		
Main activities	Production and circulation of new knowledge via networked activity, collaboration and emulation	Conduct of scientific institutions and programmes within the national system infrastructure and policy framework
Coordination and regulation	Local–national professional self-regulation on the basis of global collegial scientific norms	National law, official regulation, policy, financing systems, national cultural conventions, expectations and norms
Synergies with other science system		
People	Scientists who are global disciplinary leaders and also active in national or local scientific institutions	Scientists who are science leaders in national or institutional settings and are also active in global science
Practices	Knowledge generated or accessed in global science system triggers national funding and infrastructure support	Nationally grounded scientific capacity strengthens the capacity of scientists to participate and contribute globally

As Table 1 suggests, in material terms, global science is constituted by the work published in English language journals and codified by the two major bibliometric companies, Clarivate Analytics (Web of Science) and Elsevier (Scopus), together with the scientists themselves and the collaborative networks in which ideas are acknowledged and codified, data exchanged, joint projects funded and executed out and co-authored papers prepared. Global science is self-managed by scientists in distributed professional networks, interacting with data and publishing companies (Lariviere et al., 2015), and informally regulated by scientific protocols. National science systems are materially underpinned by nation-states, with legal, regulatory, policy and financial frameworks. They are housed in research universities and in some countries, government laboratories. Note that national science output is not simply a subset of global science. National science includes both the national part of global activity—the shared objects include global papers and also scientists and infrastructures present in both scales—and also national materials, activity and outputs outside the global circuits.

National financing, infrastructure and equipment are essential not just to national policy goals but also to global science output. In turn, global science stimulates national investment in science and access to global science provides the nation with a much larger epistemic pool. Often the same scientists and laboratories are active in both domains, though individuals vary in the extent to which they orient to the national setting and/or to the global discipline (Adams, 2013). While some international collaboration is supported by national governments, other collegial links largely by-pass governments or are unknown to them. Nevertheless, the global and national science systems each provide conditions and resources for the other: they are synergistic.

Yet the imaginaries of the two systems, as well as the associated social practices, are also profoundly different. Wagner et al. (2015) describe global science as ‘operating orthogonally to national systems’ (p. 12). National science systems are normatively centred on the nation-state, and their mission is governed by national security, prosperity and other nation-bound objectives. Global science is grounded in the professional science communities, and its mission is the production, codification and circulation of knowledge. It also tends to embody the free-wheeling autonomous faculty cultures typical of the US universities that were central to the early evolution of the Internet and through it global science. Its instrumental object is not the national economy but global problems accessible to scientific solutions. Its horizon is not the border of national jurisdiction or national interest but world society.

At the same time, relations between global and national science vary by nation. In the USA and UK, national activity blends more seamlessly with global activity, because the global system embodies Anglo-American practices and Anglo-American output has global standing. In China, Russia, Iran, Brazil and many other countries, the national language component is large and can diverge from global science in methods and topics. Global papers are a minority of total activity, especially in the social sciences (Marginson & Xu, forthcoming).

### Intersections of scales

As this suggests, higher education institutions and systems are complex assemblages in scalar terms, and intersections between scales create distinctive potentials. Movement between scales can generate opportunity and resource. Marston et al. (2005) refer to ‘scale jumping’ whereby power established in one scale is transferred to another; and cities and states that ‘rescale’ or ‘reterritorialize’ (p. 418). Universities and national systems rescale or jump scales when they merge across distances, creating multiple sites in one country, or they open international branch campuses (Wilkins & Huisman, 2012) or online distance education. Nations foster global hubs (Lee, 2015). Site-based local university corporations and national consortia establish MOOC platforms in the global scale.

Combining scales brings new or hybrid spaces into being. In a study of the International Baccalaureate, Resnik (2012) finds that where international organisations and corporations meet national-local education, this is a ‘frontier zone… neither completely national nor international’ (p. 251). These zones have two normative centres: national governance in the country of education and the IB organization with an identity outside specific nations. The ambiguity enables multiple relations and agentic strategies. Another example is institutional branch campuses that operate across national borders. These engage two national governments with a third normative centre, the mobile institution itself, creating a complex frontier zone with unstable primacy between the agents. Where multilateral organizations such as OECD and the World Bank meet national education systems, this again creates a frontier space, in which only the nation is likely to alter itself, but each party retains autonomy. Dale (2005) refers to ‘the pluri-scalar nature of educational governance’ (p. 133).

Multi-scalar vision enables new kinds of reflexivity. Institutional executives use global rankings to monitor comparative performance and status, with national-local standing in mind (Hazelkorn and Mihut, 2021). Institutions and/or academic units use benchmarking with foreign universities to lift local capability (Wang et al., 2011). In national policy-making, global templates and standards are used to evaluate national–local provision and performance, while at the same time, national objectives and values can be used to critically interrogate the global template. Both reflexivities enlarge the local and transcend its limits.

Multiple scales are also worked synergistically on the basis of a division of labour. In science, most countries use global networking and production to help build national and local activity (Olechnicka et al., 2019). Chinese research policy and institutional and researcher practice have sustained an effective ‘national/global synergy’ whereby international research collaboration and national capacity augment each other (Marginson, 2022a, 2022c). Likewise, individual universities use practices in the global scale such as research cooperation, talent recruitment and international student revenues to enhance their resources and status in the national and local scales. Reciprocally, they use national resources and status to build pan-national regional and global activity. As noted, Friedman’s (2018a) ‘New Elite’ defined itself as a ‘global university’. The local was hybridized with the global, and this strengthened the university’s national status. Yet while scale jumping often augments scope, activity and capacity overall, some institutions use scale jumping into the global to reduce their local and national responsibilities (Stein, 2017, p. 542).

The university administrators interviewed by Friedman (2018b) were nimble in combining and switching between scales. They described their elite US and UK institutions as globally open, cosmopolitan and serving the global common good. They also saw national boundaries as ‘common sense, natural and enduring’ (p. 247) and discussed students primarily in terms of national characteristics rather than culture, class or gender (p. 255). ‘Nationalized ways of talking about the world’, some ‘crude and stereotypical’ were ‘the basic discursive tools’ of personnel ‘engaged in the internationalization of academic programmes and general operations’ (p. 248). They saw their universities as ‘embodying national characteristics, and … obliged to serve national interests’ (p. 247) by generating economic activity, soft power and globally competent graduates (p. 257). The global and national missions were seen as compatible, though some interviewees stated that the main priority in the educational programme was educating national citizen students, with international students seen as a means of fostering cultural awareness at home (p. 256).

The capacity of agents to mix and match scales varies markedly. It is not so much calibrated on the basis of institutional positioning within the national-social hierarchy, as is the case with the calibration of global missions (Friedman, 2018a). It is calibrated on the basis of a global hierarchy: the national position in relation to the Anglophone hegemony in language and culture, as is the case with differences in the relations between national and global science (see above). In the Anglo-American universities, ‘the global has arisen alongside the national without displacing it’. The two can readily accommodate each other (Friedman, 2018b, p. 259). Activities in one scale blends into activity in the other. Elsewhere there are larger cross-scalar tensions. Yang (2014) notes that ‘to non-Western societies, modern universities are an imported concept’. They have spread worldwide from their Euro-American origins ‘mainly due to colonialism’. The model ‘has never been tolerant toward other alternatives, leading to the inefficacy of universities in non-Western societies, on whom a so-called “international” perspective has been imposed from the outset. What is lacking is an appropriate combination of the “international” and the local’. For example, the tension between Westernization and indigenization is obvious in China, ‘a country with a continuous history of fostering unique cultural heritages for thousands of years’ (p. 153).

Scale jumping and cross-scale complementarities work until they do not. In the decoupling of scientific relations between the USA and China after 2018, defensive national geo-politics trumped the benefits of global collaboration and national/global synergy (Lee & Haupt, 2020). Likewise, in Denmark, mobile student numbers have been reduced following induced tensions between, on one hand, global openness and English language use, on the other hand local-national sensibilities (Tange & Jaeger, 2021). Multiple scales trigger anxieties as well as opportunities. Cross-border students experience new freedoms and cultural hybridities but also loneliness (Sawir et al., 2008), displaced locality and fragmented identity.

### Primacy among scales

If scales in higher education are multiple and heterogeneous, how then to understand their configuration in relation to each other? The glonacal paper stated that the global, national and local had no fixed order of importance, though in particular cases, activity in one or another could be causal. Kosmutzky (2015) likewise argues that neither national models nor the ‘transnational level’ is necessarily framing or determining. There are ‘multiple interdependencies’. This is not universally understood. Scale-based analyses often define scales in terms of ‘hierarchical thought’ (Marston et al., 2005, p. 421) and universal not contextual scalar primacy. It is difficult to deal with a shifting causal pattern, it requires case by case investigation. It is easier to work with models that sort the world with a consistent scalar structure, with scale deployed in an essentialist manner to underpin one or another universal narrative. But this leads to errors. Like all such a priori claims, fixed scalar primacy conceals more than it reveals. Notions of fixed scalar primacy bedevil higher education studies in at least three ways.

The first form of fixed scalar primacy is a hierarchy of scales. The published diagram of the glonacal heuristic (Marginson & Rhoades, 2002, p. 291) was misleading in one respect. It implied the global, national and local scales were structural replicas, ‘scale-invariance’ (Katz & Ronda-Pupo, 2019), as if the only difference between scales was the size of the container. This in turn implied that the global scale was the supreme container and ultimate determinant, contradicting the paper’s text. It must be said that the diagram’s error is widely shared. Common visions of scale in geography and other social sciences are a cascading hierarchy of levels, such as vertical scaffolding; widening concentric circles like planetary orbits; or the identical Russian *Matryoshka* dolls of ascending size that fit into each other (Herod, 2008, pp. 226–228; Gregory et al., 2009, pp. 664–666). These spatial visions are highly misleading. They conceal fundamental differences in the nature of the scales and privilege scales that have broad scope (Marston et al., 2005, p. 427), feeding visions of bigger-smaller and outer-inner determination. This fosters over-emphasis on the causal power of large structures such as global capitalism, and tends to downplay the potentials of agency, which is equated with the allegedly subordinate local scale.

The second form of fixed scalar primacy is theorizations that acknowledge the multi-scalar setting but nevertheless assume universal determinism by one or another scale. Institutional theory permanently privileges ‘world society’, and also the institutional scale, while diminishing the agency of nations and individuals. As noted, other theorizations position the global economy as the inevitable determinant. Reviewing national policies on education in the Asia–Pacific countries, Rizvi et al., (2005) emphasize that ‘it is a mistake to assert, as many do, that reforms are a structural outcome of globalization’ (p. 12). Though OECD and World Bank norms are disseminated effectively and global corporations also wield influence, global policies are not simply adopted by national governments. Global policies also reinterpreted and ignored at national level. Valimaa (2004) discusses the assertion of national factors in Finland in the context of globalization. Nor is national agency confined to the ‘West’. Beerkens (2010) shows how global models are reconstituted in differing ways in higher education policy in Malaysia and Indonesia. Arguably, the embeddedness of institutions (especially public institutions) in national systems is a more shaping causal influence than is the global embeddedness of nations.

The third form of fixed scalar primacy is to see the world solely through the lens of one scale, rejecting scalar multiplicity altogether. This again privileges that one scale as the locus of causality. The most common lenses are methodological nationalism and methodological globalism: radical simplifications that are deployed as would-be instruments of power. ‘So many of our accustomed ways of imagining space have been attempts to tame it’ (Massey 2005, p. 152).

#### The odd couple: methodological globalism and methodological nationalism

Methodological globalism is the belief in a reified global scale ‘outside thought’ (Rizvi et al., 2005, p. 11) that determines all other scales—for example neoliberal claims that national higher education and its institutions must adjust to the inevitable transformations driven by global capitalism. An earlier example of methodological globalism, pitched against rather than in favour of global capitalism, is world-systems theory (Wallerstein, 1974). Here, nations are seen as subordinated to a fixed division of labour between ‘centre’ countries in North America and Europe, under-developed countries in the global ‘periphery’ which are doomed to perpetual dependence, and ‘semi-periphery’ countries between the two other groups. Like the neoliberal argument about economic globalization, world-systems theory sees domination by the Euro-American ‘centre’ as inevitable.

Methodological nationalism is an especially potent limiting influence in higher education studies. This is ‘the belief that the nation/state/society is the natural social and political form of the modern world’ (Wimmer & Schiller, 2003, p. 301). It rests on the ‘internalist’ fallacy that the trajectory of nations is entirely determined by their own efforts (Conrad, 2016, p. 88). Methodological nationalism is pervasive, shaping the outlook of governments, national public debate and much of social science. It is difficult to generate or to find data in any categories other than nation-bound categories (Beck, 2007), as Komotar (2021) notes in relation to higher education (p. 8). ‘Methodological nationalism operates both about and for the nation-state, to the point where the only reality we are able to comprehensively describe statistically is a national, or at best an international one’ (Dale, 2005, p. 126).

The methodological nationalist lens tends to occlude or marginalize phenomena beyond or below the nation-state. In higher education, it tends to marginalize cross-border connections and exclude global systems such as global science from view. It eliminates the potential for global responsibility at a distance (Massey, 2004). Methodological nationalism does not altogether exclude the world outside the nation, but represents it as a mosaic of separated nation-states, without either common systems or an ontology outside those nations. For example, in many studies of international collaboration in science, the networked global science system is effectively obliterated by arbitrarily splitting co-authorships between countries (Marginson, 2022c)—as if there is a meaningful *epistemic* difference between, say, French physics and Korean physics.

Methodological nationalism also shapes a permanent disjunction between global mobility and nation-state control (Massey, 2002, p. 293). Through the methodological nationalist lens, mobility within countries appears normal while mobility between countries appears as anomalous. The nation-bound imagining that people should stay where they ‘belong’ takes priority over the imaginings and practices of mobile students and the institutions that house them. Borders ‘change the balance between security and liberties … illiberal practices at border zones are embedded in ordinary politics of the liberal state’ (Basaran, 2008, p. 339). Diasporic students stay for years in the country of education but never truly arrive, occupying a grey zone in which neither home nor host country provides them with effective citizen rights (Marginson, 2012).

Methodological nationalism should not be confused with normative nationalism, which is the presumption of one or another national interest (Beck, 2007). The critique of methodological nationalism does not constitute a rejection of national identity, nor a rejection of the use of the nation or nation-state as a unit of analysis. As noted, the nation-state is central to the organization of higher education. Nation-based data are certainly needed. But recognition of the national scale does not have to carry with it the methodological exclusion of other geo-cognitive scales and their causal potentials. There are many critiques of methodological nationalism (e.g. in addition to the above, in political science, geography, sociology and social theory Harvey, 2005; Alexander, 2005, p. 81; Beck, 2007; Chernilo, 2007; in educational studies Matthews & Sidhu, 2005; Valimaa & Nokkala, 2014, among others). In ‘Beyond the “national container”’, Shahjahan and Kezar (2013) explicitly build on the glonacal paper to mount a far-reaching critique of methodological nationalism in higher education studies.

By definition, methodological globalism and methodological nationalism exclude each other. But ideology and policy narratives are not regulated by logic, and in many countries, methodological globalism and methodological nationalism have become oddly spliced together. The neoliberal narrative of the global knowledge economy in education combines the two. Massey (2005) discusses how these two ‘contradictory geographical imaginations’ are reconciled (p. 163). The knowledge economy narrative rests on a global/local binary. The global scale is external, economic and determining: global market *forces*. The nation is subordinated to this transcendent global scale. Within the national container, institutions and individuals, the agents of the local, are again reduced. They must choose from the repertoire of neo-liberal policies that the national government has designed as responses to the global imperative (Cantwell & Maldonado-Maldonado, 2009). In this way Massey’s global/local binary is implemented at two levels: local higher education is locked down simultaneously by external globalization and again by the nation-state. ‘Students are to be educated to become economic globalization’s next agents’ (Stein, 2017, p. 540). This double reduction is also pervasive in higher education studies (Altbach & Knight, 2007). For example, the widely cited argument of Knight (2003) on the definition of 'internationalization' specifically echoes the odd coupling in the knowledge economy discourse. Knight states that a transcendent external ‘globalisation is changing the world of internationalization’, while ‘internationalization’, which is the world understood from within the national container rather than in the global scale, ‘is changing the world of education’ (p. 3). 

Shahjahan (2019) further explores this scalar determinism. In affective terms, capitalist globalization fosters ‘anxieties and aspirations’ that capture the agency of persons in higher education and bind them to the nation. Colonized in this way, agents cannot see the world as a whole. This ‘precludes a planetary consciousness, as we are stuck in global discourses underpinned by nation-state categories and identities’ (Shahjahan & Grimm, 2022, p. 9). Strikingly, the neo-liberal scalar structure, with its fault line between the externalized global economy and nation-bound agency and politics, is *exactly* that imagined by nativist populism. Neoliberalism proffers a solution to the tension between globalization and local agency: global capital takes priority. Populism simply ramps up the tension without limit.

Methodological globalism and methodological nationalism in higher education are flatly contradictory. Only when scale is imagined as successive *Matryoshka* dolls of diminishing size can they be reconciled: in the *Matryoshka* formula, the outer methodological globalism absolutely determines methodological nationalism, and that in turn absolutely controls the educational agents within. The neoliberal imaginary is ‘inconsistent, falsely self-evident, never universalizable, but powerful’ (Massey, 2005, p. 87).

### An open ontology of scale

How then does space-making play out in the absence of a fixed scalar order? First, agents vary in their practices of leading scale. For some local agents, national valuations and relations are primary, as with Friedman’s (2018b) university administrators. For other local agents, global relations may come first, such as those scientists who are focused primarily on global disciplinary networks. Second, social practices and relations are subject to a complex mix of changing scalar drivers. Third, scalar primacy is always contextually articulated by time and place. If the global scale is the locus of causation today, it may not be tomorrow. Likewise the national scale.

Take cross-border student mobility (Marginson et al., 2010), which is shaped at the intersection of global convergence and national borders and by the intersection between global flows and international relations. While in a more global era ‘the state may have less control over ideas … it remains a controller of its borders and the movement of people across them’ (Hirst & Thompson, 1995, p. 420). The framing of cross-border education as nation-determined international education varies on the basis of national political and educational cultures. At the same time, everywhere, a variable range of global, regional, national, city-based and institutional drivers affect the supply of and demand for places (e.g. see OECD, 2016, pp. 328–345). There are no permanent lines of causation in cross-border student mobility but many causal factors. These vary between nations and between student families. In commercial countries like the UK, nation and institution are driven to expand places, though institutional capacity and national migration policy set limits. In supply countries with soft power or cross-cultural educational objectives (e.g. policies in Japan that focus on the internationalization of home country students), there may be less incentive to expand raw numbers. In Europe, regional drives to integrate professional labour markets and ground common identities have underpinned large scale mobility in the Erasmus programme (Brooks, 2018). Student demand for cross-border education is affected by national system capacity. Demand for education perceived to be of good quality that is unmet locally can send students abroad. That student demand is also affected by the labour markets for returning graduates. Scalar primacy can shift quickly, as when the global flow of Chinese graduate students into the USA was slowed in the late 2010s by US national security concerns.

## Conclusions

The glonacal paper (Marginson & Rhoades, 2002) was positioned between two extreme propositions: higher education is being totally transformed by economic globalization, and higher education is continuing as before. This played out well after 2002, when global activity in higher education continued to grow while the nation-state did not fade away. As the glonacal paper argued, the global and national scales were not in a zero-sum trade-off. Most of the propositions in the glonacal paper have retained their explanatory power: the open ontology and agentic potentials; the multiple characters of scale; the strategic importance of intersections and combinations between scales; the refusal of spatial uniformity and all fixed scalar determinations including those based on claims about the ‘imperatives’ of the global economy; and, always, the need for contextualized research and analysis.

Despite higher education's multiple constituencies and agendas, its prestige economy, its historical grounding and its embededdness in states, all of which block its wholesale reduction to capitalist production (Marginson, 2013), by 2022 the sector had moved closer to the global knowledge economy imaginary than the glonacal paper had expected. The 2002 paper underestimated two related elements. First, the extent to which global higher education would be analogised as a global economic market, a mode of thought that was powerfully advanced by the global rankings which began in 2003–2004 (though the market idea is more strongly held in the Anglosphere and East Asia than elsewhere). Second, the continued potency of methodological nationalism, despite its theoretical illogic, the visible facts of the multi-scalar sector, and the expanding global flows of knowledge in a connected higher education world. In the eyes of most people, the nation-state is still the boundary of society and vision. The global scale is seen as external in relation to the sector, despite its co-penetration of all other scales. This constrains spatial imagining and practices. The knowledge economy myths are locked in by ideology and the nexus between institutions and national government. Dale (2005) points to the ‘embedded statism’ of the sector (p. 124). This positions higher education at the centre of society, which is especially attractive to many institutions *qua* institutions, but it overshadows the sector’s potential autonomy.

The present paper has reviewed, illustrated and expanded upon the glonacal insights. It has theorised agentic space-making in higher education as an always-emerging unity of materiality, imagining and social practices. It has explored the way agents in higher education form, use, open and close space. It has taken further the inquiry into the multiple geo-cognitive scales in higher education and pinpointed the pathologies of scalar determinism, especially methodological nationalism. There is much scope to further explore, test and develop the ideas presented here in empirical studies of higher education.

This theorization of space and scale also has many implications for understanding relations of power in the sector. It is apparent throughout the paper that space and scale are experienced as both irreducibly diverse in the horizontal sense and highly unequal (though not inevitably so) in the vertical sense. While the paper is focused on space and scale rather than on relations of power per se, the paper’s examples indicate that space-making is not only joined to power but is itself an act of power. Agents’ scope for effective action in space-making is calibrated on the basis of their economic and cultural resources, the location of their organisations and systems within positional hierarchies and their relation to the cultural-linguistic hegemony of the Anglophone zone (for more discussion see Marginson, 2022d). At the same time, emerging higher education systems and agents pursue decolonial and dehegemonizing strategies that entail new kinds of spaces and connections. The local material resources essential to agency are accumulated, new ideas and combined activities and endogenous social practices are developed, national agency is grounded and advanced, and more autonomous global strategies are pursued. Space and scale are always changing, and no structural hierarchy is fixed in place forever.

Space is one of the primary coordinates of the higher education world. It is continually made and remade in encounters between agents. However, the single-scale visions and scale-driven universals must be cleared away to bring a fuller geography of higher education to life. ‘There are no rules of space and place’, states Massey (2005). What matters is the social relations that constitute, and are constituted by, each spatial configuration. Setting aside scalar determinism, and all other social laws based on an iron-bound pre-given structure, allows the fuller scope of agency to emerge. What is actual in society is real, and what is possible is also real. Relational space is the incubator not just of multiple existing life story trajectories but of the new and future intersecting trajectories and shared zones in higher education. Space in its differing and overlapping scales is an inexhaustible resource that humans make for themselves and the medium of their slowly expanding freedoms.

## Footnotes

1 Because the present paper reviews the 2002 glonacal paper from a critical distance, personal pronouns (‘we said’ etc.) are not used. The glonacal paper’s assumptions have largely held up, but this author would now do it somewhat differently, as the present paper indicates.

2 On 25 October 2022, there were 384 citations of Marginson and Rhoades (2002) in Web of Science and 1301 in Google Scholar. Other studies of education that parallel the glonacal spatiality, with an open ontology and multi-scalar approach, include Matthews and Sidhu (2005), Ishikawa (2009), Resnik (2012) and Friedman (2018a; 2018b).

3 The role of ranking in shaping competitive global relations in higher education is a large topic but see, among others, Marginson (2007), Ishikawa (2008), Shahjahan et al. (2017) and Lloyd and Ordorika (2021). On the back of their publicly provided rankings data, QS and Times Higher Education mount commercial services that assist institutions to perform within the competitive global market the rankers have institutionalized.

4 Brooks (2018) suggests that some UK institutions focus on global citizenship and employability because in contrast with other English-speaking countries, UK provides limited opportunities for mobile students to migrate.

## References

[CR1] Adams J (2013). The fourth age of research. Nature.

[CR2] Alexander J (2005). “Globalization” as collective representation: The new dream of a cosmopolitan civil sphere. International Journal of Politics, Culture and SocIety.

[CR3] Altbach P, Knight J (2007). The internationalization of higher education: Motivations and realities. Journal of Studies in International Education.

[CR4] Anderson, B. (2006). *Imagined Communities: Reflections on the origins and spread of nationalism*. Revised Edition. Verso.

[CR5] Appadurai A (1990). Disjuncture and difference in the global cultural economy. Theory, Culture & Society.

[CR6] Appadurai, A. (1996). *Modernity at large: Cultural dimensions of globalization*. University of Minnesota Press.

[CR7] Appadurai, A. (1999). Globalization and the research imagination. *International Social Science*, *51*(160), 229–238. Wiley Online Library. 10.1111/1468-2451.00191

[CR8] Appadurai, A. (2014). Arjun Appadurai. *Globalizations*, *11*(4), 481–490 10.1080/14747731.2014.951209

[CR9] Archer M (2005). Structure, agency and the internal conversation.

[CR10] Archer, M. (1995). *Realist social theory: The morphogenetic approach.* Cambridge University Press.

[CR11] Archer, M. (2000). *Being human: The problem of agency.* Cambridge University Press.

[CR12] Basaran T (2008). Security, law, borders: Spaces of exclusion. International Political Sociology.

[CR13] Bayly, C. (2004). *The birth of the modern world, 1780–1914: Global connections and comparisons*. Wiley-Blackwell.

[CR14] Beck U (2000). The cosmopolitan perspective: Sociology of the second age of modernity. British Journal of Sociology.

[CR15] Beck U (2007). The cosmopolitan condition: Why methodological nationalism fails. Theory, Culture and Society..

[CR16] Beck U, Grande E (2010). Varieties of second modernity: The cosmopolitan turn in social and political theory and research. The British Journal of Sociology.

[CR17] Beerkens E (2010). Global models for the national research university: Adoption and adaptation. Globalisation, Societies and Education.

[CR18] Beerkens, E. (2004). *Global opportunities and institutional embeddedness: Higher education consortia in Europe and Southeast Asia.* Doctoral dissertation, University of Twente. https://www.utwente.nl/en/bms/cheps/phd-page/cheps-alumni-and-their-theses/thesisbeerkens.pdf. Accessed 15 Oct 2022

[CR19] Bourdieu P (1984). Distinction: A social critique of the judgement of taste.

[CR20] Brooks R (2018). Higher education mobilities: A cross-national European comparison. Geoforum.

[CR21] Calhoun C (2003). ‘Belonging’ in the cosmopolitan imaginary. Ethnicities.

[CR22] Cantwell B, Maldonado-Maldonado A (2009). Four stories: Confronting contemporary ideas about globalisation and internationalization in higher education. Globalisation, Societies and Education.

[CR23] Castells, M. (2000). *The rise of the network society*. Volume I of *The information age: Economy, society and culture* (2^nd^ ed.). Blackwell.

[CR24] Cerny P (1997). Paradoxes of the competition state: The dynamics of political globalization. Government and Opposition.

[CR25] Chernilo, D. (2007). *A social theory of the nation state: The political forms of modernity beyond methodological nationalism*. Routledge.

[CR26] Chinchilla-Rodríguez Z, Sugimoto C, Larivière V, Bornmann L (2019). Follow the leader: On the relationship between leadership and scholarly impact in international collaborations. PLoS One.

[CR27] Chou M-H, Ravinet P (2017). Higher education regionalism in Europe and Southeast Asia: Comparing policy ideas. Policy and Society.

[CR28] Conrad S (2016). What is global history?.

[CR29] Cox, R. (1981). Social forces, states and world orders: Beyond international relations theory. *Millenium: Journal of International Studies*, *10*(2), 126–155. 10.1177/03058298810100020501

[CR30] Cummings, W. (2006). *Globalization and higher education: Reflection on international trends.* Keynote speech for 2006 Hong Kong Comparative Education Society. [unpublished]

[CR31] Dale, R. (2005). Globalisation, knowledge economy and comparative education. *Comparative Education*, *41*(2), 117–149. http://www.jstor.org/stable/30044528. Accessed 14 Oct 2022

[CR32] Deem R (2001). Globalisation, new managerialism, academic capitalism and entrepreneurialism in universities: Is the local dimension still important?. Comparative Education.

[CR33] Enders J (2004). Higher education, internationalisation and the nation-state: Recent developments and challenges to governance theory. Higher Education.

[CR34] Frank D, Meyer J (2007). University expansion and the knowledge society. Theory and Society.

[CR35] Friedman J (2018). The global citizenship agenda and the generation of cosmopolitan capital in British higher education. British Journal of Sociology of Education.

[CR36] Friedman J (2018). Everyday nationalism and elite research universities in the USA and England’. Higher Education.

[CR37] Giddens, A. (1986). *The constitution of society: Outline of the theory of structuration*. University of California Press.

[CR38] Goddard J, Vallance P (2013). The University and the City..

[CR39] Gregory, D., Johnston, R., Pratt, G., Watts, M. & Whatmore, S. (eds.) (2009). *The dictionary of human geography*. 5^th^ edition. Wiley-Blackwell.

[CR40] Guan J, Zhang J, Yan Y (2015). The impact of multilevel networks on innovation. Research Policy.

[CR41] Gupta A, Tesluk P, Taylor MS (2007). Innovation at and across multiple levels of analysis. Organization Science.

[CR42] Harvey, D. (1990). Between space and time: Reflections on the geographical imagination^1^. *Annals of the Association of American Geographers,**80*, 418–434. 10.1111/j.1467-8306.1990.tb00305.x. Accessed 14 Oct 2022

[CR43] Harvey, D. (2005). The sociological and geographical imaginations. *International Journal of Politics, Culture and Society*, *18*, 211–255. http://www.jstor.org/stable/20059684

[CR44] Hazelkorn, E. & Mihut, G. (eds.) (2021). *Research handbook on university rankings: Theory, methodology, influence and impact*. Edward Elgar.

[CR45] Hazelkorn, E. (2020). Evolving architecture of/for international education and global science. In K. Godwin & H. de Wit (eds.), Intelligent internationalization: The shape of things to come 19–22. 10.1163/9789004418912_003

[CR46] Held D, McLew A, Goldblatt D, Perraton J (1999). Global transformations: Politics, economics and culture.

[CR47] Hennemann S, Rybski D, Liefner I (2012). The myth of global science collaboration—Collaboration patterns in epistemic communities. Journal of Informetrics.

[CR48] Henry M, Lingard R, Rizvi F, Taylor S (2001). The OECD, globalisation and education policy.

[CR49] Herod A, Holloway S, Rice S, Valentine G, Clifford N (2008). Scale: The local and the global. Key concepts in geography.

[CR50] Hirst P, Thompson G (1995). Globalization and the future of the nation state. Economy and Society.

[CR51] Horta H (2009). Global and national prominent universities: Internationalisation, competitiveness and the role of the State. Higher Education.

[CR52] Ishikawa M (2009). University rankings, global models, and emerging hegemony: Critical analysis from Japan. Journal of Studies in International Education.

[CR53] James P, Steger M, Robertson R, Gulmez DB (2016). Globalization and global consciousness: Levels of connectivity. Global culture: Consciousness and connectivity.

[CR54] Jones G (2008). Can provincial universities be global institutions? Rethinking the institution as the unit of analysis in the study of globalisation and higher education. Higher Education Policy.

[CR55] Jones J, Woodward K, Marston S (2007). Situating flatness. Transactions of the Institute of British Geographers.

[CR56] Katz J, Ronda-Pupo G (2019). Cooperation, scale-invariance and complex innovation systems: A generalization. Scientometrics.

[CR57] Knight J (2003). Updating the definition of internationalization. International Higher Education.

[CR58] Komotar M (2021). Comparative higher education research in times of globalisation of higher education: Theoretical and methodological insights. European Educational Research Journal.

[CR59] Kosmützky A (2015). In defence of international comparative studies. On the analytical and explanatory power of the nation-state in international comparative higher education research. European Journal of Higher Education.

[CR60] Larivière V, Haustein S, Mongeon P (2015). The oligopoly of academic publishers in the digital era. PLoS One.

[CR61] Larsen M, Beech J (2014). Spatial theorizing in comparative and international education research. Comparative Education Review.

[CR62] Lee, J. [Jenny] & Haupt, J. (2020). Winners and losers in US-China scientific research collaborations. *Higher Education, 80*(1), 57–74.10.1007/s10734-019-00464-7

[CR63] Lee, J. [Jenny] & Li, X. (2022). *Racial profiling among scientists of Chinese descent and consequences for the U.S. scientific community*. Report. https://www.jstor.org/stable/40241677,https://www.committee100.org/wp-content/uploads/2021/10/C100-Lee-Li-White-Paper-FINAL-FINAL-10.28.pdf. Accessed 14 Oct 2022

[CR64] Lee, J. [Jack] (2015). The regional dimension of education hubs: Leading and brokering geopolitics. *Higher Education Policy*, *28*(1), pp. 69-89. 10.1057/hep.2014.32

[CR65] Lee, J. [Jenny] & Stensaker, B. (2021). Research on internationalization and globalization in higher education – Reflections on historical paths, current perspectives and future possibilities. *European Journal of Education,**56*, 157–168. 10.1111/ejed.12448

[CR66] Lefebvre, H. (1991). *The production of space*. D. Nicholson-Smith (Tran.). Blackwel

[CR67] Liu, H. & Metcalfe, A. (2016). Internationalizing Chinese higher education: A glonacal analysis of local layers and conditions. *Higher Education, 71*(3), 399–413. http://link.springer.com/article/10.1007/s10734-015-9912-8. Accessed 14 Oct 2022

[CR68] Lloyd M, Ordorika I, Stack M (2021). International university rankings as cultural imperialism: Implications for the global South. Global university rankings and the politics of knowledge.

[CR69] Marginson S (2006). Dynamics of national and global competition in higher education. Higher Education.

[CR70] Marginson S (2012). Including the other: Regulation of the human rights of mobile students in a nation-bound world. Higher Education.

[CR71] Marginson S (2013). The impossibility of capitalist markets in higher education. Journal of Education Policy.

[CR72] Marginson S (2022). ‘All things are in flux’: China in global science. Higher Education.

[CR73] Marginson S (2022). What drives global science? The four competing narratives. Studies in Higher Education.

[CR74] Marginson S (2022). Global science and national comparisons: Beyond bibliometrics and scientometrics. Comparative Education.

[CR75] Marginson S (2022). What is global higher education?. Oxford Review of Education.

[CR76] Marginson S, Rhoades G (2002). Beyond national states, markets, and systems of higher education: A glonacal agency heuristic. Higher Education.

[CR77] Marginson S, Nyland C, Sawir E, Forbes-Mewett H (2010). International student security.

[CR78] Marginson, S. & Xu, X. (forthcoming). Hegemony and inequality in global science: Problems of the center-periphery model. Accepted by *Comparative Education Review*, 4 January 2022

[CR79] Marginson, S. (ed.) (2007). *Prospects of higher education: Globalization, market competition, public goods and the future of the university*. Sense Publishers

[CR80] Marston S, Smith N (2001). States, scales and households: Limits to scale thinking? A response to Brenner. Progress in Human Geography.

[CR81] Marston S, Jones J, Woodward K (2005). Human geography without scale. Transactions of the Institute of British Geographers.

[CR82] Massey D (2002). Globalisation: What does it mean for geography?. Geography.

[CR83] Massey D, May S (2003). Some times of space. Olafur Eliasson: The weather project.

[CR84] Massey D (2004). Geographies of responsibility. Geografiska Annaler.

[CR85] Massey D (2005). For space.

[CR86] Matthews J, Sidhu R (2005). Desperately seeking the global subject: International education, citizenship and cosmopolitanism. Globalisation, Societies and Education.

[CR87] Mearman A (2005). Sheila Dow’s concept of dualism: Clarification, criticism and development. Cambridge Journal of Economics.

[CR88] Naidoo R, Unterhalter E, Carpentier V (2010). Global learning in the neo-liberal age: Implications for development. Global inequalities in higher education: Whose interests are we serving?.

[CR89] Odora Hoppers, C. (2009). Education, culture and society in a globalizing world: Implications for comparative and international education. Compare, 39(5), 601–614. 10.1080/03057920903125628

[CR90] Olechnicka A, Ploszaj A, Celinska-Janowicz D (2019). The geography of scientific collaboration.

[CR91] Oleksiyenko A (2019). Academic collaborations in the global marketplace.

[CR92] Organisation for Economic Cooperation and Development (OECD) (2016). *Education at a glance 2016*. OECD

[CR93] Organisation for Economic Cooperation and Development (OECD) (2021). *Education at a glance 2021*. OECD.

[CR94] Organisation for Economic Cooperation and Development (OECD) (2022). *Science and technology indicators.* https://stats.oecd.org/Index.aspx?DataSetCode=MSTI_PUB. Accessed 16 October

[CR95] Pieterse J (2020). Global culture 1990, 2020. Theory, Culture and Society.

[CR96] Pieterse, J. (1995). Globalization as hybridization. In M. Featherstone, R. Robertson & S, Lash (eds.) 1995. *Global Modernities* 45–68. Sage. 10.4135/9781446250563.n3

[CR97] Powell, J., Baker, D., & Fernandez, F. (eds.) (2017). *The century of science: The global triumph of the research university*. Vol. 33. International Perspectives on Education and Society. Emerald Publishing Limited. 10.1108/S1479-3679201733

[CR98] Resnik, J. (2012). The denationalisation of education and the expansion of the International Baccalaureate. *Comparative Education Review*, *56*(2), 248–269. http://www.jstor.org/stable/10.1086/661770

[CR99] Rizvi F (2005). Identity, culture and cosmopolitan futures. Higher Education Policy.

[CR100] Rizvi F (2011). Experiences of cultural diversity in the context of an emergent transnationalism. European Educational Research Journal.

[CR101] Rizvi, F., Engel, L., Nandyala, A., Rutkowski, D. & Sparks, J. (2005). *Globalization and recent shifts in educational policy in the Asia Pacific: An overview of some critical issues*. APEID UNESCO. https://unesdoc.unesco.org/ark:/48223/pf000015296 . Accessed 15 Oct 2022

[CR102] Rizvi, F., Lingard B., & Rinne, R. (eds.) (2022). *Reimagining globalization and education*. Routledge.

[CR103] Rizvi, F. (2006). *Epistemic virtues and cosmopolitan learning*. Radford Lecture, Adelaide, 27 November. https://files.webservices.illinois.edu/3943/rizvi___proofepistemicvirtues.pdf. Accessed 15 Oct 2022

[CR104] Roberston, S., Olds, K., Dale, R. & Dang, Q. (eds.) (2016). *Global regionalism and higher education: Projects, processes and politics*. Edward Elgar.

[CR105] Roberston, S., Rosenzvaig, M. & Maber, E. (2023). Globalisation, culture and higher education. In G. Yair (ed.) *Research Handbook on Culture and Education*. Edward Elgar

[CR106] Robertson S (2006). Absences and imaginings: The production of knowledge on globalisation and education. Globalisation, Societies and Education.

[CR107] Robertson, R. (1992). *Globalization: Social theory and global culture*. Sage

[CR108] Santos, B. (2007). Beyond abyssal thinking: From global lines to ecologies of knowledges. Review (Fernand Braudel Center), 30(1), 45–89. https://www.jstor.org/stable/40241677. Accessed 12 Oct 2022

[CR109] Sassen, S. (ed.) (2002). *Global networks, linked cities*. Routledge.

[CR110] Sawir E, Marginson S, Deumert A, Nyland C, Ramia G (2008). Loneliness and international students – An Australian study. Journal of Studies in International Education.

[CR111] Sayer A (2000). Realism and social science.

[CR112] Schofer E, Meyer J (2005). The worldwide expansion of higher education in the twentieth century. American Sociological Review.

[CR113] Shahjahan R (2019). From ‘geopolitics of being’ towards inter-being: Envisioning the ‘in/visibles’ in the globalization of higher education. Youth and Globalization.

[CR114] Shahjahan R, Grimm A (2022). Bringing the ‘nation-state’ into being: Affect, methodological nationalism and the globalisation of higher education. Globalisation, Societies and Education.

[CR115] Shahjahan R, Kezar A (2013). Beyond the ‘national container’: Addressing methodological nationalism in higher education research’. Educational Researcher.

[CR116] Shahjahan R, Ramirez GB, Andreotti V (2017). Attempting to imagine the unimaginable: A decolonial reading of global university rankings. Comparative Education Review.

[CR117] Sheppard E, McMaster B (2004). Scale and geographic inquiry: Nature, society and method.

[CR118] Sidhu R (2005). Building a global schoolhouse: International education in Singapore. Australian Journal of Education.

[CR119] Soja E (2010). Cities and states in geohistory. Theory and Society.

[CR120] Stein S (2017). The persistent challenges of addressing epistemic dominance in higher education: Considering the case of curriculum internationalization. Comparative Education Review.

[CR121] Tange H, Jaeger K (2021). From Bologna to welfare nationalism: International higher education in Denmark, 2000–2020. Language and Intercultural Communication.

[CR122] Taylor, C. (2003). *Modern social imaginaries*. Duke University Press.

[CR123] Teferra, D. (2019). Defining internationalisation – Intention versus coercion. *University World News*, 23 August. https://www.universityworldnews.com/post.php?story=20190821145329703

[CR124] The Economist (2022). The tricky restructuring of global supply chains. https://www.economist.com/leaders/2022/06/16/the-tricky-restructuring-of-global-supply-chains. Accessed 15 Oct 2022

[CR125] Torres C, Schugurensky D (2002). The political economy of higher education in the era of neoliberal globalization: Latin America in comparative perspective. Higher Education.

[CR126] Valimaa J (2004). Nationalisation, localisation and globalisation in Finnish higher education. Higher Education.

[CR127] Valimaa J, Nokkala T (2014). The dimensions of social dynamics in comparative studies on higher education. Higher Education.

[CR128] van der Wende, M. (2002). Higher education globally: Towards new frameworks for research and policy. In *The CHEPS Inaugurals 2002,* 29–69. Twente University Press

[CR129] Vaughan-Williams N (2008). Borders, territory, law. International Political Sociology.

[CR130] Wagner, C., Park, L., & Leydesdorff, L. (2015). The continuing growth of global cooperation networks in research: A conundrum for national governments. W. Glanzel (ed.). *PLOS One* 10 (7): e0131816. 10.1371/journal.pone.0131816. 10.1371/journal.pone.0131816 PMC4510583 26196296

[CR131] Wallerstein I (1974). The rise and future demise of the world capitalist system: Concepts for comparative analysis. Comparative Studies in Society and History.

[CR132] Wang QH, Wang Q, Liu N, Altbach P, Salmi J (2011). Building world-class universities in China: Shanghai Jiao Tong University. The road to academic excellence: The making of world-class research universities.

[CR133] Watkins J (2015). Spatial imaginaries research in geography: Synergies, tensions, and new directions. Geography Compass.

[CR134] Wilkins S, Huisman J (2012). The international branch campus as transnational strategy in higher education. Higher Education.

[CR135] Wimmer A, Schiller N (2003). Methodological nationalism and beyond: State building, migration and the social sciences. Global Networks.

[CR136] Woodward K, Jones J, Marston S (2012). The politics of autonomous space. Progress in Human Geography.

[CR137] Xu X, Marginson S, Xu X (2022). Internationalisation of Chinese humanities and social sciences. Changing higher education in East Asia.

[CR138] Yang R (2014). China’s strategy for the internationalisation of education. Frontiers of Education in China.

[CR139] Yang R, Suter L, Smith E, Denman B (2019). Riddled with gaping wounds: A methodological critique of comparative and international studies in education: Views of a professor. The SAGE Handbook of Comparative Studies in Education.

[CR140] Yang, L., Marginson, S. & Xu, X. (2022). Thinking through the world: A *tianxia* heuristic for higher education. *Globalisation, Societies and Education*, 10.1080/14767724.2022.2098696

